# Prognostic and predictive significance of tumor length in patients with esophageal squamous cell carcinoma undergoing radical resection

**DOI:** 10.1186/s12885-016-2417-8

**Published:** 2016-07-07

**Authors:** Jie Wu, Qi-Xun Chen

**Affiliations:** Department of Thoracic Surgery, Zhejiang Cancer Hospital, 1 East Banshan Road, Hangzhou, 310022 Zhejiang Province China

**Keywords:** Esophageal cancer, Squamous cell carcinoma, Tumor length, Prognosis

## Abstract

**Background:**

The objective of this study was to investigate the prognostic and predictive significance of tumor length in patients with esophageal squamous cell carcinoma undergoing radical resection.

**Methods:**

Tumor length and other clinicopathological variables were retrospectively evaluated in 1435 patients with squamous cell carcinoma treated with radical resection between 2003 and 2010. Tumor length was analyzed as categorical and continuous variable. Associations with overall survival were assessed with Cox proportional hazards models. Model-based nomograms were constructed. Predictive accuracy was measured with C-index. Decision curve analysis was used to evaluate clinical usefulness of prediction models.

**Results:**

Both categorically and continuously coded tumor length were independent prognostic factors in multivariable analysis. Adding categorically and continuously coded tumor length to TNM staging model increased predictive accuracy by 0.2 and 0.4 % respectively. Decision curve analysis revealed that the models built by the addition of categorically or continuously coded tumor length did not perform better than TNM staging model.

**Conclusions:**

Tumor length is an independent prognostic factor in patients with esophageal squamous cell carcinoma treated with radical resection. It increases predictive accuracy of TNM staging system for overall survival in these patients. But it does not increase clinical usefulness of TNM staging system as a prediction model.

**Electronic supplementary material:**

The online version of this article (doi:10.1186/s12885-016-2417-8) contains supplementary material, which is available to authorized users.

## Background

Esophageal cancer is one of the most aggressive malignancies throughout the world with the sixth highest cancer deaths annually [1]. The tumor, node, metastasis (TNM) staging system is an important tool to assess prognosis, guide therapy, formulate treatment protocols and promote the exchange of information between different centers [2]. In the current 7th edition of American Joint Committee on Cancer (AJCC) TNM staging system, histological grading, tumor location as well as depth of esophageal wall invasion are used for stage grouping for squamous cell carcinoma [3]. Recently some authors found tumor length was an independent prognostic factor for esophageal cancer [4–11], and even suggested incorporating tumor length into TNM staging system to identify high-risk patients for postoperative therapy [4–9]; while others did not find any associations between tumor length and long-term survival in patients with esophageal cancer [12–15]. Therefore the prognostic role of tumor length still needs to be ascertained. On the other hand, whether incorporating tumor length into TNM staging system could generate a better prediction model for outcomes of esophageal cancer patients also requires to be further investigated. The purpose of this study was to evaluate the prognostic and predictive significance of tumor length in patients with esophageal squamous cell carcinoma treated with radical resection within a single institution.

## Methods

### Study population

**Fig. 1:**
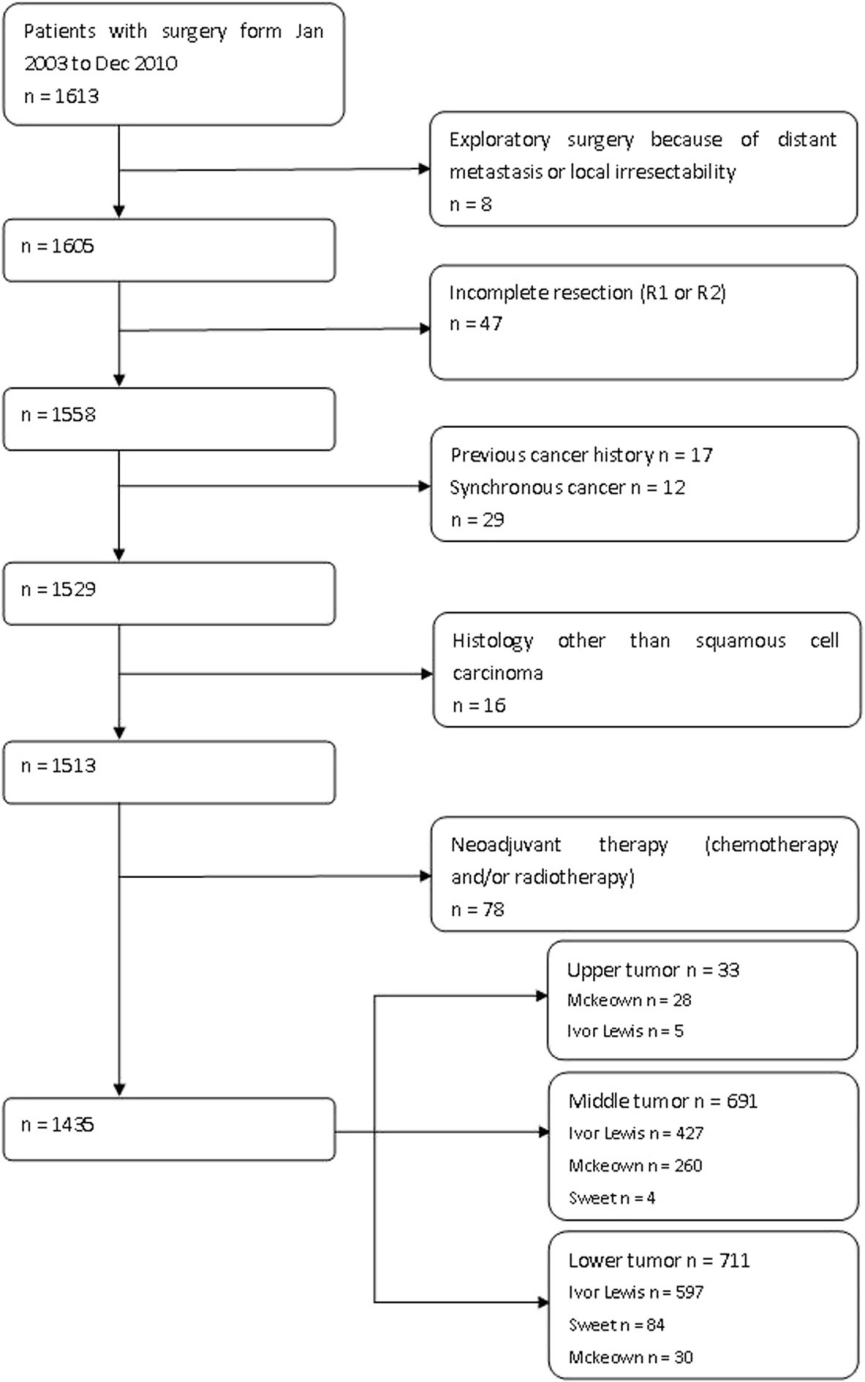
Flow chart of study population

This study was approved by the institutional review board of Zhejiang Cancer Hospital and the need for individual patient consent was waived. The study was conducted with data collected from a prospectively collected database for esophageal cancer. Between January 2003 and December 2010, 1613 consecutive cases were surgically treated at the Department of Thoracic Surgery of Zhejiang Cancer Hospital. Because an institutional electronic medical record system was used in our hospital since January 2003, this date was chosen as the starting date for the study. A total of 1435 patients with esophageal squamous cell carcinoma after resection with curative intent were included in this study (Fig. 1). Among 47 patients excluded because of incomplete resection, 35 patients had macroscopic residual disease (R2 resection) and 12 patients had microscopic disease (R1 resection: positive proximal resection margin in nine cases and positive distal resection margin in three cases). Seventeen patient with previous cancer history (gastric cancer in eight cases, lung cancer in four cases, laryngeal caner in three cases, breast cancer in one cases and malignant lymphoma in one cases) were excluded. Of 12 patients excluded because of synchronous cancer, seven patients had synchronous gastric cancers, three patients had synchronous hypopharyngeal cancers, one patient had a synchronous laryngeal cancer, and one patient had synchronous leukemia. Sixteen patients with non-squamous carcinoma (adenocarcinoma in six cases, adenosquamous carcinoma in four cases, small cell carcinoma in four cases, and carcinosarcoma in two cases) were also excluded. Because neoadjuvant therapy may influence postoperative pathological staging and tumor length, patients with neoadjuvant therapy were excluded. All of these 1435 patients received preoperative evaluations including endoscopy with biopsy, barium swallow examination, computerized tomography of the chest and upper abdomen, and ultrasound of the neck. Pulmonary and cardiac function tests were routinely performed to assess medical operability. Recurrent laryngeal nerve palsy and the presence of clinical supraclavicular or cervical nodal involvement were considered a contraindication for surgery. Histological diagnosis of each of the patients was established before treatment. Written informed consents were obtained from all patients before surgery.

### Surgical procedure

Three surgical approaches were commonly used: Ivor Lewis procedure, cervico-thoraco-abdominal approach (Mckeown prodcedure), and left thoracotomy approach (Sweet procedure). Ivor Lewis procedure and Sweet procedure with anastomosis in the chest apex were usually performed when the tumor located in the lower and middle segment of the esophagus. When the tumor located in the middle or upper segment of the esophagus, Mckeown procedure with anastomosis in the left neck was mainly conducted (Fig. 1). Meanwhile, the choice of surgical procedure also depended on surgeons’ preferences. Two-field (mediastinal and upper abdominal) lymph node dissection was routinely performed for all patients. The extent of mediastinal lymph node dissection included all nodal tissue associated with esophagus in the chest from the superior mediastinal nodes and nodes along both recurrent laryngeal nerves to the hiatus. The extent of upper abdominal lymph node dissection included the paracardial, lesser curvature, left gastric, common hepatic, celiac, and splenic nodes. Three-field (cervical, mediastinal and upper abdominal) lymph node dissection was not routinely performed. However, this procedure was also performed selectively by surgeons depending on their preference. The extent of cervical lymph node dissection included supraclavicular and cervical paraesophageal nodes.

### Pathological examination

After surgical resection, the esophageal specimen was opened longitudinally from proximal to distal, extending this incision along greater curve of stomach if attached. The anatomical locations of the removed nodes were labeled by the operating surgeon. All specimens were fixed in 10 % formalin overnight, unpinned. and then sent to pathological examination. Tumor length was measured to the closest to 1 mm. In addition to tumor length, pathological details including histology type, differentiation, depth of invasion, lymph node status, vascular invasion, perineural involvement, the number of resected lymph nodes as well as proximal and distal surgical resection margin were reported. Circumferential resection margin was not routinely examined at our institution. Data from pathological reports were reviewed retrospectively. All patients were restaged based on the 7th edition of the American Joint Committee on Cancer TNM staging system [3].

### Follow-up

In general, a follow-up examination was performed in our outpatient department every 3 months for the first 2 years and 6 months thereafter. The routine follow-up examination included a physical and routine blood examinations, blood chemistry, measurement of tumor markers (carcinoembryonic antigen, squamous cell carcinoma antigen), radiograph of the chest, and ultrasound. Computed tomography of the chest and upper abdomen were done every 6 months. Endoscopy was done yearly. Survival time was defined as the period from the date of surgery till death (including surgical death and non-cancer related death) or the most recent follow-up. The duration of follow-up ranged from 1 to 128 months (mean 29.8 months, median 24.0 months).

### Statistical analysis

The normally distributed continuous data were described as mean ± standard deviation. Categorical data were describes as counts and proportions. Continuous variables were compared by student *t* test. The Pearson Chi-square test was used to compare categorical variable. The survival time was calculated by the Kaplan-Meier method, and the log rank test was used to assess the differences in survival between groups. To determine an ideal cutoff value for tumor length, the relationship between tumor length and death from esophageal cancer was investigated by using a scatter plot of the variable versus Martingale residuals from a Cox proportional hazard regression model without the variable of interest. A smoothed line fit of the scatter was then applied to detect the ideal cutoff value [16]. Based on the cutoff value, the tumor length could be treated as a categorical variable. Univariable Cox regression models were fitted to assess the relative effect of categorically and continuously coded tumor length and other clinicopathological variables on overall survival. The predictive accuracy of each clinicopathological variable was determined and was defined as the ability to discriminate between patients who died from cancer. The predictive accuracy was assessed with Harrell's concordance index (C-index) [17], which is an approximation of area under curve for time-to-event data. A C-index of 0.5 is equal to chance discrimination and a C-index of 1.0 represents a perfect discrimination. Multivariable Cox proportional hazards models were fitted to identify independent prognostic factors. A backward procedure based on the Akaike Information Criterion (AIC) was used for variable selection.

The parameters of the TNM staging system for esophageal squamous cell carcinoma (T stage, N stage, Grade and Location) were selected as a multivariable base model. Predictive accuracy of the TNM staging base model was then compared on the addition of tumor length. Multivariate regression coefficients of the predictive variables were used to develop nomogarms. Model performance was internally validated by measuring both discrimination and calibration [17]. Discrimination was evaluated by C-index as mentioned previously. Calibration was performed by a calibration curve, in which predicted versus actual survival are graphically depicted. Both discrimination and calibration were evaluated on this cohort using bootstrapping with 200 resamples [17]. To assess the clinical usefulness of prediction models, decision curve analysis was used by visualizing the net benefits of prediction models when different threshold probabilities were considered [18, 19].

For all statistical tests, two sided *P* < 0.05 was regarded as statistically significant. All statistical analyses were performed using SPSS version 17.0 (SPSS, Chicago, IL), and R software version 3.1.3 (https://www.r-project.org/).

## Results

### Cutoff value of tumor length and patients characteristics

**Fig. 2:**
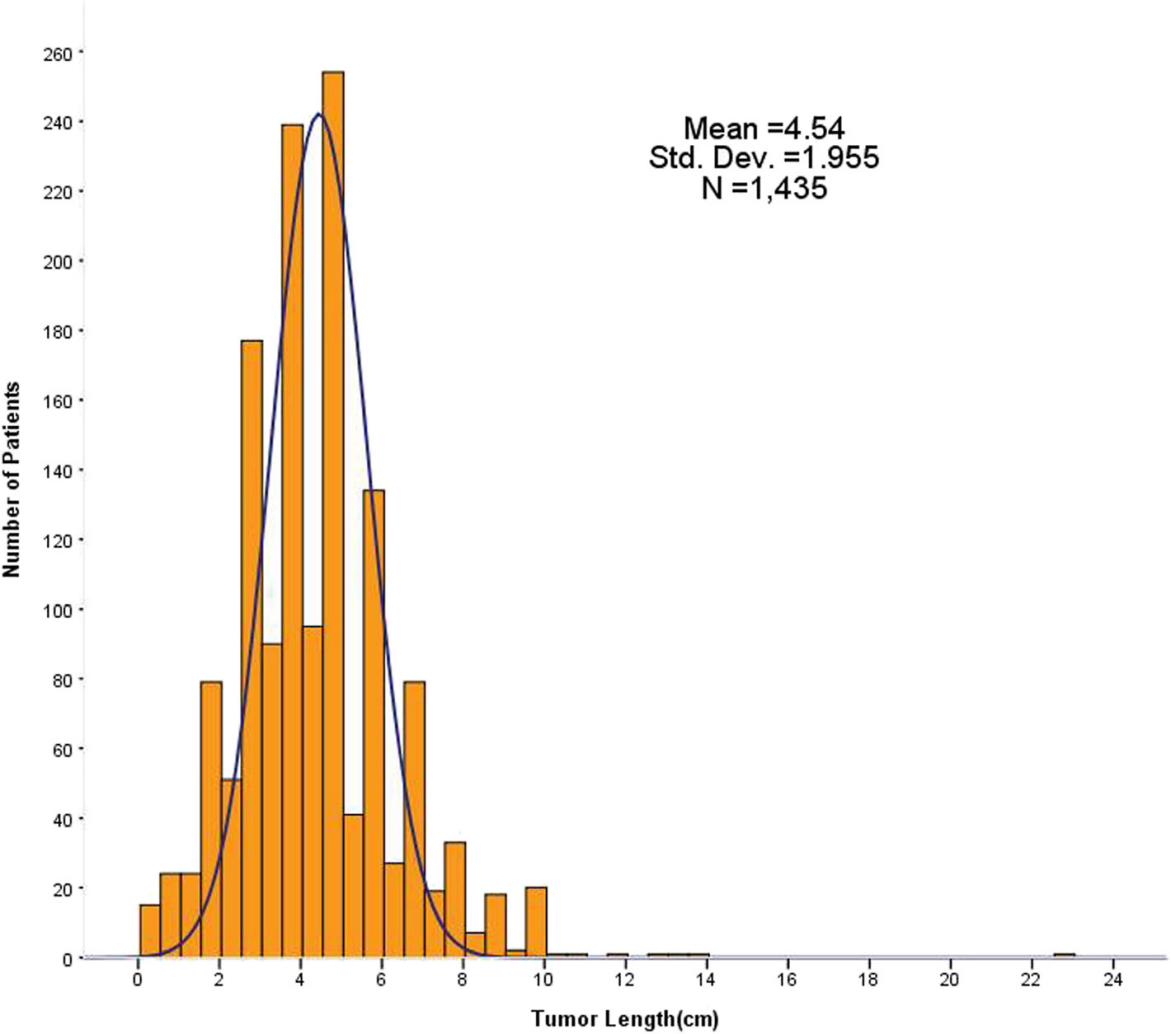
Histogram of tumor length for the entire cohort of 1435 patients

**Fig. 3:**
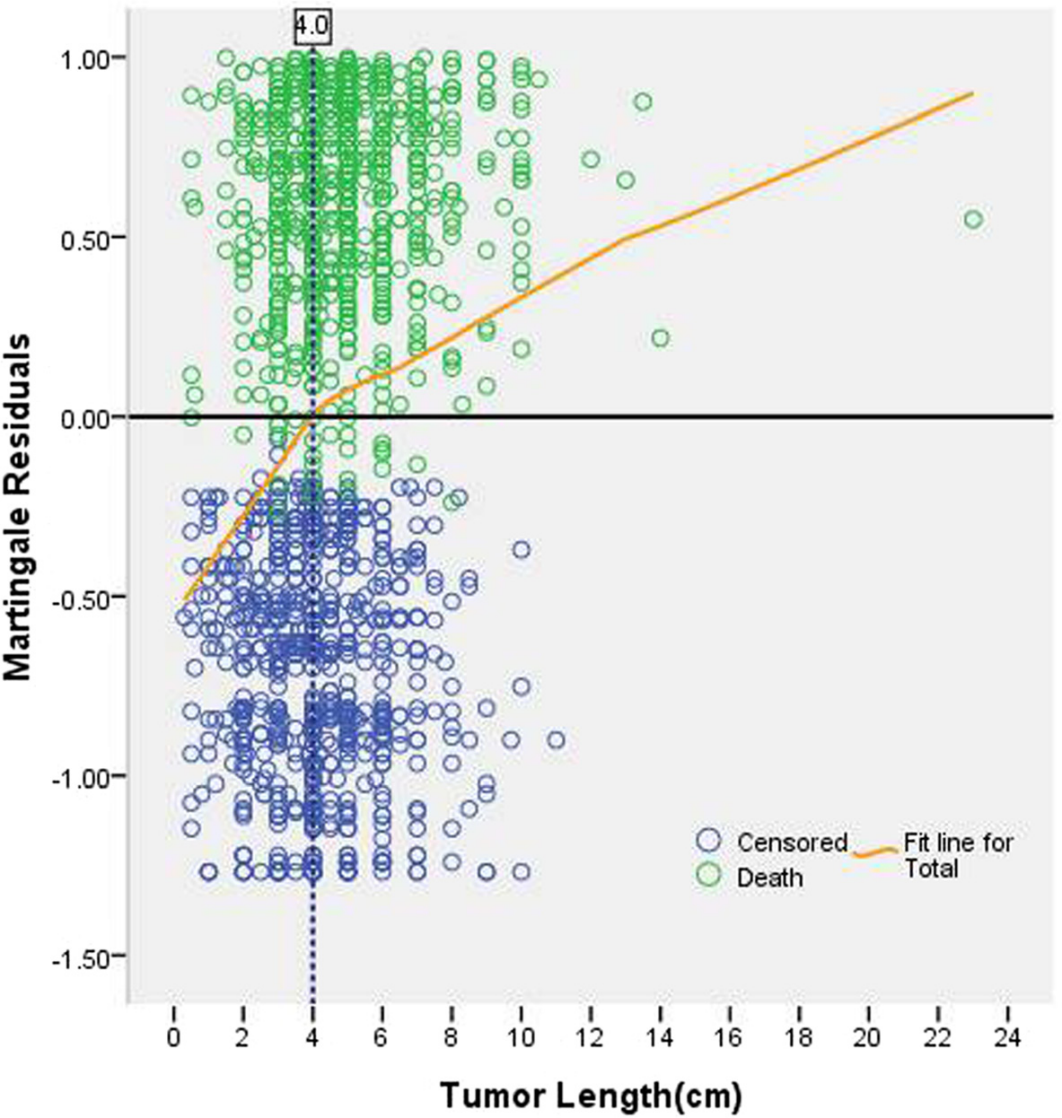
Scatter plot of tumor length versus Martingale residuals for the entire cohort of 1435 patients. Patients above the horizontal line (zero) were at increased risk for death, and those below were at decreased risk for death compared with the expected risk from Cox proportional hazard regression model. Curved line represents scatterplot smoother. Point at which smoother line cross horizontal line occurs at 4 cm, indicating this would be an ideal cutoff value of tumor length for these patients

Tumor length ranged from 0.3 to 23.0 cm (mean, 4.5 cm; median, 4.5 cm). The frequency distribution of tumor length for the entire cohort patients was shown in Fig. 2. Martingale residuals suggested 4 cm was an ideal cutoff value for tumor length (Fig. 3). On the basis of this cutoff value, patients were then divided into two groups (≤4 cm versus > 4 cm). Comparison of clinicopathological characteristics between these two groups was shown in Table 1. Tumor length > 4 cm significantly correlated with younger age (*P* = 0.023), male (*P* < 0.001), lower location (*P* = 0.01), increasing T stage (*P* < 0.001), worse N stage (*P* < 0.001), and more resected lymph nodes (*P* < 0.001), whereas no association with differentiation, vascular invasion, and perineural involvement could be found.

**Table 1:** Relationship between tumor length and other clinicopathological characteristics

Variable	Tumor length		*P* value
	≤4.0 cm	>4.0 cm	
All cases	699	736	
Age (year)	58.7	57.7	0.023
Sex			<0.001
Male	585	669	
Female	114	67	
Tumor location			0.01
Upper	22	11	
Middle	364	327	
Lower	313	398	
Differentiation			0.910
G1	107	107	
G2	131	137	
G3	461	492	
T stage			<0.001
T1	166	21	
T2	150	93	
T3	342	540	
T4	41	82	
N stage			<0.001
N0	376	295	
N1	182	193	
N2	106	170	
N3	35	78	
Vascular invasion			0.429
No	578	620	
Yes	121	116	
Perineural involvement			0.160
No	576	585	
Yes	123	151	
Number of resected lymph nodes	24.0	26.5	<0.001

### Univariable and multivariable analysis

**Fig. 4:**
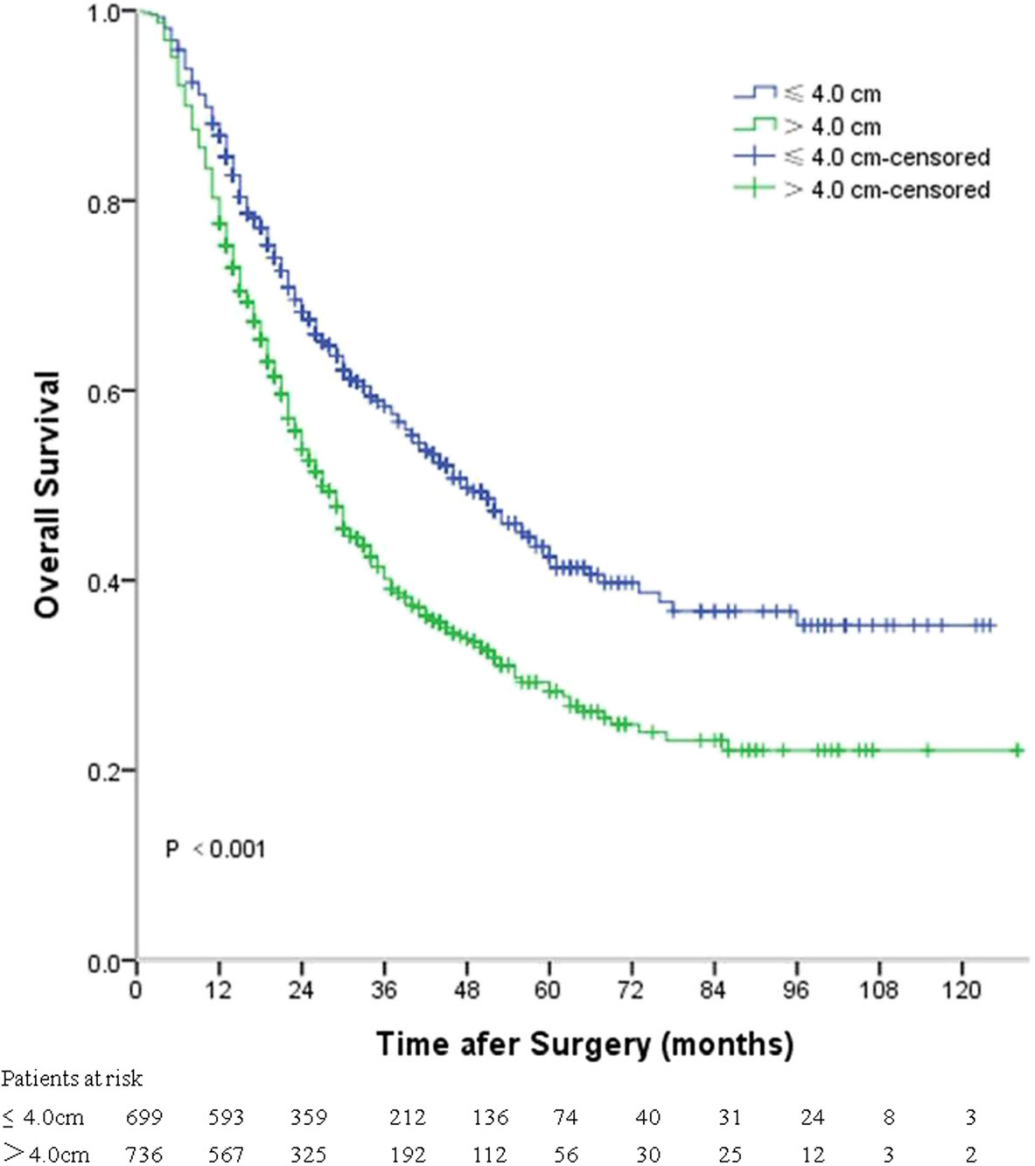
Kaplan-Meier curves depicting overall survival according to tumor length

Univariable analysis identified both categorically (*P* < 0.001) and continuously (*P* < 0.001) coded tumor length were significant prognostic factors for overall survival (Table 2). The median survival time for patients with tumor length ≤ 4 cm was 48 months (95 % CI 40.8–55.2 months), whereas for those with tumor length > 4 cm it was 27 months (95 % CI 24.3–29.7 months) (*P* < 0.001) (Fig. 4). Other significant prognostic factors included sex (*P* = 0.025), differentiation (*P* < 0.001), T stage (*P* < 0.001), N stage (*P* < 0.001), vascular invasion (*P* = 0.038), and perineural involvement (*P* < 0.001) (Table 2). To assess predictive accuracy for each clinicopathological variable, C-index was calculated. Among all of the clinicopathological variables, tumor length was found to be the third best predictor (58.1 % as a continuous variable, 56.1 % as a categorical variable) after N stage (67.1 %) and T stage (60.5 %) (Table 2).

**Table 2:** Univariable analysis of overall survival in 1435 patients according to clinicopathological variables

Variable	HR	95 % CI	*P* value	(C-index) (%)
Age	1.007	0.998–1.015	0.118	52.2
Sex				51.6
Male (reference)	1			
Female	0.766	0.608–0.966	0.025	
Tumor location				53.2
Upper (reference)	1			
Middle	1.318	0.758–2.292	0.328	
Lower	1.534	0.882–2.666	0.129	
Differentiation				55.4
G1 (reference)	1			
G2	2.094	1.622–2.703	<0.001	
G3	1.442	1.154–1.802	<0.001	
T stage				60.5
T1 (reference)	1			
T2	2.253	1.572–3.230	<0.001	
T3	3.499	2.545–4.809	<0.001	
T4	5.593	3.835–8.156	<0.001	
N stage				67.1
N0 (reference)	1			
N1	2.001	1.661–2.409	<0.001	
N2	3.640	3.007–4.406	<0.001	
N3	6.180	4.882–7.823	<0.001	
Vascular invasion				51.5
No (reference)	1			
Yes	1.224	1.001–1.482	0.038	
Perineural involvement				53.8
No (reference)	1			
Yes	1.595	1.345–1.891	<0.001	
Number of resected lymph nodes	1.002	0.995–1.008	0.577	50.3
Tumor length				56.1
≤ 4.0 cm (reference)	1			
> 4.0 cm	1.582	1.368–1.830	<0.001	
Tumor length*	1.121	1.088–1.155	<0.001	58.1

*tumor length treated as a continuous variable

In Cox multivariate analysis, variable selection based on backward method using AIC was preformed. Both categorically (*P* = 0.018) and continuously (*P* < 0.001) coded tumor length were independent prognostic factors for overall survival. Other independent prognostic factors included age, differentiation, T stage, N stage, and number of resected lymph nodes. Sex, tumor location, vascular invasion and perineural involvement did not have significant impact on overall survival (Table 3).

**Table 3:** Multivariable analysis of overall survival in 1435 patients according to clinicopathological variables

	Categorical tumor length			Continuous tumor length		
Variable	HR	95 % CI	*P* value	HR	95 % CI	*P* value
Age	1.011	1.002–1.019	0.014	1.010	1.002–1.019	0.015
Male (reference)	1		0.371			
Female	0.896	0.706–1.139		0.908	0.715–1.155	0.342
Upper tumor (reference)	1					
Middle tumor	1.150	0.660–2.005	0.622	1.169	0.671–2.038	0.581
Lower tumor	1.113	0.637–1.945	0.708	1.130	0.647–1.974	0.668
G1 (reference)	1					
G2	1.503	1.153–1.959	0.003	1.516	1.163–1.976	0.002
G3	1.184	0.942–1.488	0.148	1.180	0.939–1.483	0.157
T1 (reference)	1					
T2	1.708	1.182–2.649	0.004	1.666	1.153–2.406	0.006
T3	2.140	1.521–3.009	<0.001	2.081	1.483–2.919	<0.001
T4	2.781	1.855–4.171	<0.001	2.691	1.797–4.028	<0.001
N0 (reference)	1					
N1	1.746	1.442–2.116	<0.001	1.716	1.416–2.078	<0.001
N2	2.974	2.422–3.652	<0.001	2.961	2.412–3.636	<0.001
N3	5.128	3.969–6.626	<0.001	5.091	3.939–6.580	<0.001
Vascular invasion	1.113	0.917–1.350	0.278	1.108	0.913–1.344	0.299
Perineural involvement	1.091	0.912–1.305	0.343	1.098	0.918–1.314	0.307
Number of resected lymph nodes	0.987	0.980–0.994	<0.001	0.987	0.980–0.994	<0.001
Tumor length	1.201	1.032–1.403	0.018	1.064	1.026–1.103	<0.001

### Model comparisons

**Fig. 5:**
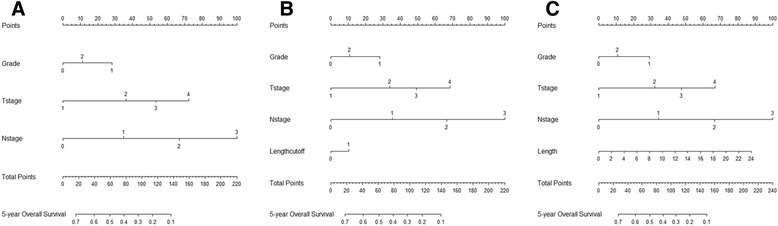
Nomograms based on Cox models to predict 5-year overall survival. **a** TNM base model; (**b**) model combining TNM parameters with categorically coded tumor length; (**c**) model combining TNM parameters with continuously coded tumor length. Instructions: The nomogram allows the users to obtain 5-year overall survival probability corresponding to a patient's clinicopathological characteristics. Locate the patient's characteristic on the variable row and draw a vertical straight up to the points row to assign a value of points for the variable. Add up the total points and drop a vertical line from the total points row to obtain 5-year overall survival

**Fig. 6:**
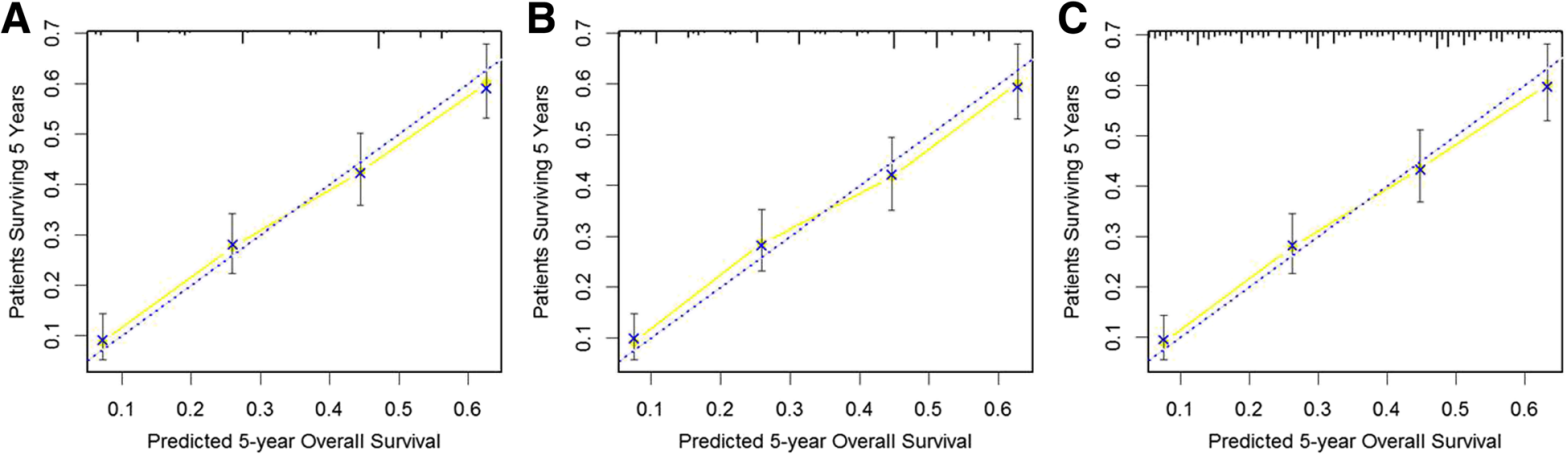
Calibration curves for internal validation of nomograms predicting 5-year overall survival. **a** TNM base model; (**b**) model combining TNM parameters with categorically coded tumor length; (**c**) model combining TNM parameters with continuously coded tumor length. The x axis nomogram-predicted probability of overall survival, and the y axis is actual survival. The diagonal line is the reference line indicating perfect calibration. The solid line indicates performance of the current nomogram

**Fig. 7:**
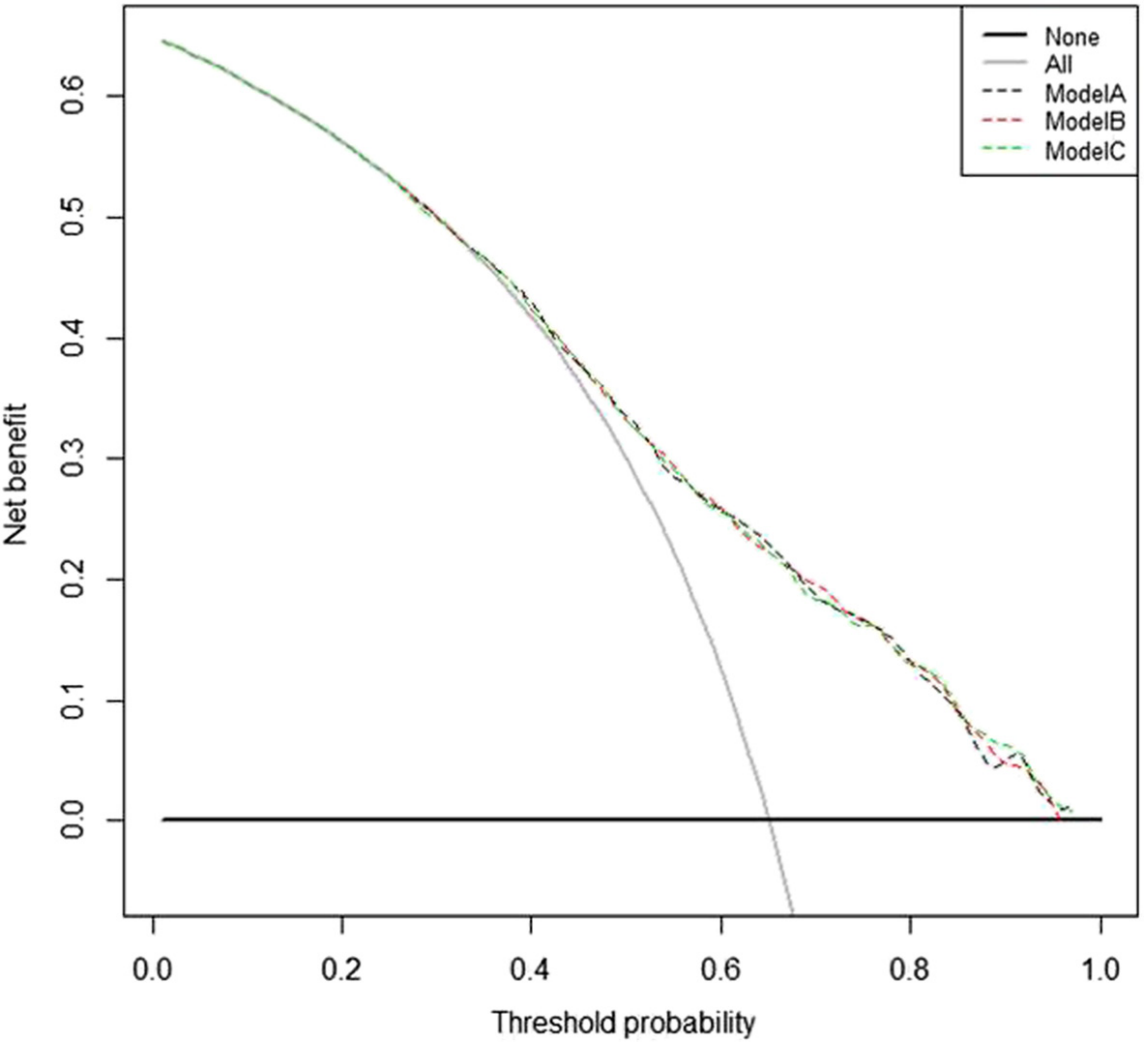
Decision curve analysis. The y axis measures net benefit, calculated by summing the benefits (true positive findings) and subtracting the harms (false positive findings). The grey line is the net benefit for a strategy of treating all patients. The horizontal line is the net benefit of treating no patients. Dotted line represents net benefit of using a new model. Model A, TNM base model; Model B, the model combining TNM parameters with categorically coded tumor length; Model C, the model combining TNM parameters with continuously coded tumor length

Three prediction models were built. The first was a TNM staging base model. The second and the third were added categorically coded and continuously coded tumor length to the base model respectively. Results of three multivariate regression models were listed in Table 4. Differentiation, T stage, and N stage were independent prognostic factors in each of the three models. Both categorically and continuously coded tumor length reached statistical significance. Tumor location did not reach statistical significance in each of the three models. Three nomograms were developed for predicting overall survival based on beta coefficients in associated models (Fig. 5). Model performance was evaluated by internal validated by bootstrapping. The bootstrap-corrected C-index for TNM staging base model was 69.4 %. The addition of categorically and continuously coded tumor length to the TNM staging base model led to an increased bootstrap-corrected C-index of 69.6 and 69.8 %, respectively. The calibration curves of the three prediction models were shown in Fig. 6. Each calibration curve showed good agreement between predicted and actual outcomes. In the decision curve analysis, three models performed similarly across a wide range threshold probabilities. Models including tumor length (either categorically or continuously coded) did not show any net benefit for predicting overall survival compared to the TNM staging base model (Fig. 7).

**Table 4:** Cox regression models for predicting overall survival

	Base model			Categorical tumor length			Continuous tumor length		
Variable	HR	95 % CI	*P* value	HR	95 % CI	*P* value	HR	95 % CI	*P* value
G1 (reference)	1			1			1		
G2	1.551	1.193–2.018	0.001	1.548	1.190–2.013	0.001	1.563	1.202–2.034	<0.001
G3	1.191	0.950–1.495	0.130	1.182	0.942–1.484	0.148	1.178	0.939–1.478	0.157
Upper tumor (reference)	1			1			1		
Middle tumor	1.192	0.684–2.075	0.535	1.174	0.674–2.044	0.571	1.188	0.682–2.068	0.543
Lower tumor	1.180	0.677–2.056	0.560	1.148	0.658–2.001	0.627	1.156	0.664–2.015	0.608
T1 (reference)	1			1			1		
T2	1.757	1.220–2.531	0.002	1.685	1.167–2.433	0.043	1.636	1.033–2.362	0.008
T3	2.296	1.651–3.193	<0.001	2.133	1.522–2.990	<0.001	2.067	1.478–2.892	<0.001
T4	3.066	2.070–4.544	<0.001	2.869	1.924–4.270	<0.001	2.772	1.864–4.123	<0.001
N0 (reference)	1			1			1		
N1	1.717	1.418–2.079	<0.001	1.725	1.425–2.089	<0.001	1.700	1.404–2.057	<0.001
N2	2.822	2.304–3.457	<0.001	2.791	2.278–3.418	<0.001	2.777	2.268–3.402	<0.001
N3	4.705	3.670–6.032	<0.001	4.654	3.629–5.968	<0.001	4.618	3.601–5.923	<0.001
Tumor length				1.170	1.005–1.363	0.043	1.058	1.020–1.097	0.002
Bootstrap-corrected C-index (%)	69.4			69.6			69.8

## Discussion

Tumor length was demonstrated as an independent prognostic factor for esophageal squamous cell carcinoma in this study. This result is in agreement with some previous studies [4–11]. But previous studies did not address the predictive role of tumor length. Accurate prediction of cancer prognosis is based on prediction models rather than on a variable alone. The current TNM staging, as a gold standard classification system to predict prognosis in patients [2], is naturally the best option for establishing a base prediction model. Although it is possible that a significant variable in multivariable modeling might not improve discrimination compared with a multivariable base model, in this cohort, the addition of tumor length did indeed increase predictive accuracy of TNM staging base model. Categorically and continuously coded tumor length increased discrimination of TNM staging base model from 69.4 to 69.6 and 69.8 % respectively. However, improved discrimination is not sufficient for a prediction model to be clinically useful [19]. In decision curve analysis, three models resulted in similar net benefits for prediction of overall survival, which suggested inclusion of tumor length did not increase clinical usefulness of TNM staging system as a prediction model.

Different methods used for deciding cutoff value of tumor length led to different cutoff values reported in published series, ranging from 2 to 5 cm [4, 5, 7–9, 11, 12, 14, 15]. Compared to those methods, Martingale residuals method used in this report might be more scientific because it comprehensively allows for clinicopathological characteristics that may impact overall survival [16]. There were also various types of tumor length used in historical literature, such as pre-operative endoscopic tumor length [4, 10], tumor length of fresh specimen measured in operation [5], and pathological tumor length measured after operation [9, 12]. Tumor length may vary depending on different measuring methods. Previous research also has demonstrated shrinkage of tumor specimen after formalin fixation [5, 9]. Here pathological tumor length was used for patients undergoing radical resection because, among all types of tumor length, it reflected the most accurate measurement and the minimal observed variation [9, 12].

Tumor location has been included in the current staging system for esophageal squamous cell carcinoma [3]. In the present study, however, tumor location was not an independent prognostic factor. Many studies focusing on prognosis of esophageal squamous cell carcinoma had similar findings too [4, 5, 20], which supports omitting tumor location as a parameter in the current TNM staging system. It is noteworthy that the number of resected lymph nodes was an independent prognostic factor in multivariable analysis. Number of resected lymph nodes has been emphasized for its prognostic significance by many scholars recently [12, 21, 22]. Particularly in node negative patients number of resected lymph nodes not only guarantees the quality of esophageal resection, but also provides accurate staging and better prognosis.

There are a few limitations of this study. First this study is limited to its retrospective nature in spite of data collected prospectively. Therefore these results need to be further confirmed by a prospective study to provide a better conclusion. Second, using different surgical procedures and different types of lymphadenctomy unavoidably leads to a certain selection bias. Third, measuring errors may exist in the process of pathological examination. Finally, although bootstrap method is used for internal validation of prediction models to obtain unbiased estimates, external validation is still needed to determine whether it can be applied to other patient groups.

## Conclusions

In conclusion, tumor length is an independent prognostic factor in patients with squamous cell carcinoma undergoing radical resection. It increases predictive accuracy of the current TNM staging system for overall survival. But it does not increase the clinical usefulness of TNM staging system as a prediction model.

## Abbreviations

AIC, akaike information criterion; AJCC, American Joint Committee on Cancer; C-index, Harrell's concordance index; TNM, tumor, node, metastasis

## Additional file

Additional file 1: Datasets supporting the conclusions of this article. (XLSX 172 kb)
